# The impact of overall radiotherapy treatment time and delay in initiation of radiotherapy on local control and distant metastases in gastric cancer

**DOI:** 10.1186/1748-717X-9-81

**Published:** 2014-03-23

**Authors:** Viacheslav Soyfer, Ravit Geva, Michael Michelson, Moshe Inbar, Einat Shacham-Shmueli, Benjamin W Corn

**Affiliations:** 1 Tel Aviv University, 6 Weizman street, 64239 Tel Aviv, Israel

**Keywords:** Stomach, Radiation, Time, Recurrence

## Abstract

**Objectives:**

To study the impact of time factors on local and distant metastases in stomach cancer.

**Methods:**

67 patients with gastric cancer who received adjuvant treatment were reviewed for the time to initiation of radiotherapy, overall duration of RT and the events of first local recurrence or distant metastasis.

**Results:**

The risk probability of local recurrence is increased by 10% (HR = 1.1, p = 0.0009) in association with each additional day of radiotherapy and by 3.8% (HR = 1.038, p = 0.13) per increased day of waiting time before the initiation of RT. The risk probability of distant recurrence was associated with an increase of 7.4% (HR = 1.074 p = 0.0031) with each additional day of RT time and by 2.3% (HR = 1.023, p = 0.0598) following the increase of a day of waiting time. Each day of prolongation of RT beyond 36 days was associated with an increased risk of local recurrence by 10% (OR = 1.1, p = 0.015). Prolongation of waiting time prior to initiation of irradiation retained significance in multivariate analysis.

**Conclusion:**

There is an association between total treatment time and, to some extent, the time between the surgery and the initiation of radiation on local control and distant metastases.

## Background

The effect of prolonged overall treatment time of radiation therapy as well as the impact of delaying the initiation of radiation treatment have been extensively discussed in the literature [1-7]. In general, it is thought that accelerated repopulation of the tumor cells during these protracted intervals is responsible for the inferior results of the treatment [1]. Increased local failures were detected after a prolonged course of radiotherapy in head and neck malignancies, lung cancer and bladder cancer [4,8,9]. We sought to describe the relationship between the interval before the initiation of radiation and overall radiotherapy time upon local control and distant metastases.

## Methods

Sixty-seven patients with primary gastric cancer were treated with adjuvant chemoradiation after curative surgery in 2003–2008 according to the protocol guidelines of INT0116 [10]. There were 31 women and 36 men. Median age was 59.8 (range 18–79) years. Patient characteristics are presented in Table 1. Median follow up was 60 months. Complete data were available on 49 patients. A total of sixty-six patients completed the prescribed adjuvant treatment between the years 2003 and 2008. One patient did not wish to continue radiation therapy after two fractions and declined to give a reason for his decision. Chemotherapy was administered in compliance with the regimen specified in INT-0116 with insufficient deviation to cause concern for confounding of our conclusions.

**Table 1:** Patient and tumor characteristics (N = 67)

**Characteristic**	**No. of patients**	**%**
Sex	36	54
Male	31	46
Female		
Primary sites		80
Proximal	54	20
Distal	13	
Pathology differentiation		
Well	4	6
Moderate	11	16
Poor	46	69
Unknown	6	9
Age, years		
Median	59.8	
Range	18-78	
Stage		
1	1	1.5
2	40	60
3	17	25
Unknown	9	13.5
Lymph Nodes status		
N0-1 (0–6)	39	58
N2 (7–15)	12	18
N3 (>15)	7	10
Unknown	9	14

The mean treatment time was 37 days (range 8–80) SD 10.8 days). The mean time to initiation of RT was 93.8 days (SD24.5). The total radiation treatment time and the interval from surgery to the initiation of radiation were each correlated with the rate of local recurrence and distant metastases. The secondary goal of the study was to characterize the relationship between the time to appearance of the first local or distant metastasis as a function of overall treatment time and the period between the surgery and radiotherapy.

Regression analysis of survival data based on the Cox proportional hazards model was used to explain the effect of time to the initiation of RT and overall RT time on local and distant recurrence hazard rates. The patients were grouped in days of RT or days to the initiation of RT in accordance with their proportional representation in the study population. The distribution of overall treatment time was as follows: less than 36 days (57.1%) and greater than or equal to 36 days (42.9%). Regarding the time to initiation of RT the distribution was as follows: less than or equal to 76 days (first 25%), 76-107(50%) and above 107 days (upper 25% of population). Logistic regression analysis was applied to reveal the odds ratio of local recurrence and distant metastases as a function of time to initiation of treatment and overall radiotherapy time.

We also applied Cox regression analysis to reveal the possible interaction between the observed results of timing and recurrence with other co factors: age, number of involved lymph nodes and stage of the disease.

Approval was obtained from the institutional review board of Tel Aviv Medical Center to carry out this analysis.

## Results

### Documentation of relative intervals

For the five patients who experienced local recurrence and the 44 who did not, the median total radiotherapy treatment time was 52.6 days, (range, 32–80; SD 24.7) and 34.8 days (range 31–56 SD 5 days), respectively (HR 1.09, 95% CI p- 0.01). With regards to the same comparative sub-groups, the time from surgery to initiation of radiation therapy was 108.3 days (range 88–142; SD 29.4) versus 88.4 days, (range 24–146; SD 23.3), respectively (HR 1.04, 95% CI p- 0.134).

### Impact on distant failure

For the 19 patients who experienced distant metastases and the 30 individuals who did not, the total radiotherapy time was 38.1 days (range 31–80; SD 12.4) versus 35.6 days (range 32–79; SD 8.6), respectively (HR 1.07, p- 0.003). With respect to the same comparative sub-groups, the time from surgery to the initiation of radiation therapy was 93.8 days (SD 23.6) versus 87 days (SD 24.1), respectively (HR 1.02, p- 0.059).

### Impact on local recurrence

**Figure 1:**
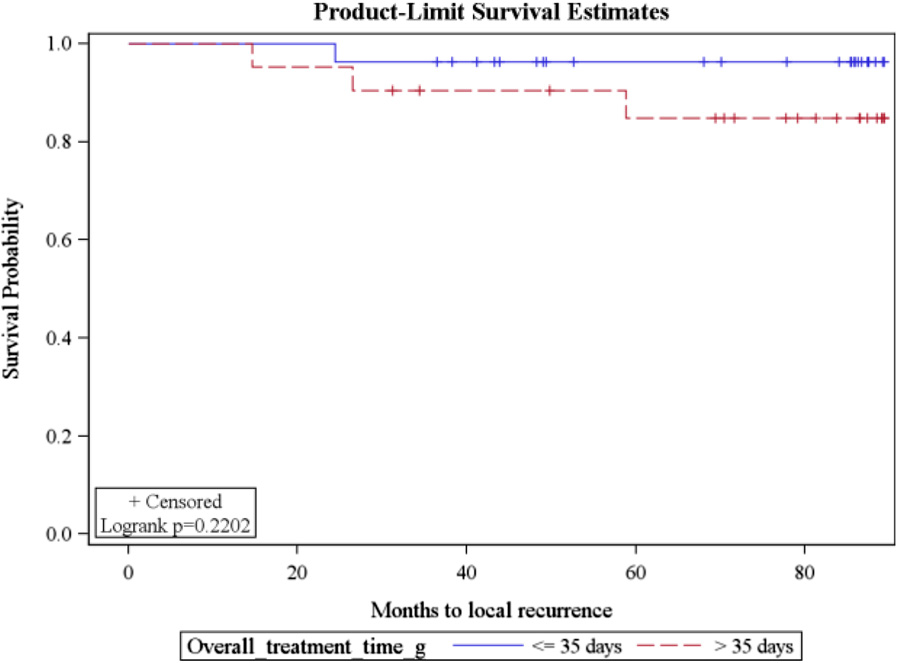
Kaplan Meier curves of time to local recurrence (comparison of 3 groups of time to treatment).

Cox regression analysis showed that each additional day that the course of irradiation was extended beyond the projected time, was associated with an increased risk probability of local recurrence by 10% (HR = 1.1 (1.04-1.016), p =0.0009). An increase of a day in the duration between surgery and the initiation of radiation was associated with an increased (albeit non-significantly) risk probability of local recurrence by 3.8% (HR = 1.038 (0.989-1.09), p = 0.13) (Table 2, Figure 1).

**Table 2:** Local recurrence (Cox regression analysis)

**Parameter**	**P value**	**Hazard ratio**	**95% hazard ratio confidence limits**	
Duration of RT days	0.0009	1.101	1.040	1.165
Waiting time	0.1341	1.038	0.989	1.090

**Figure 2:**
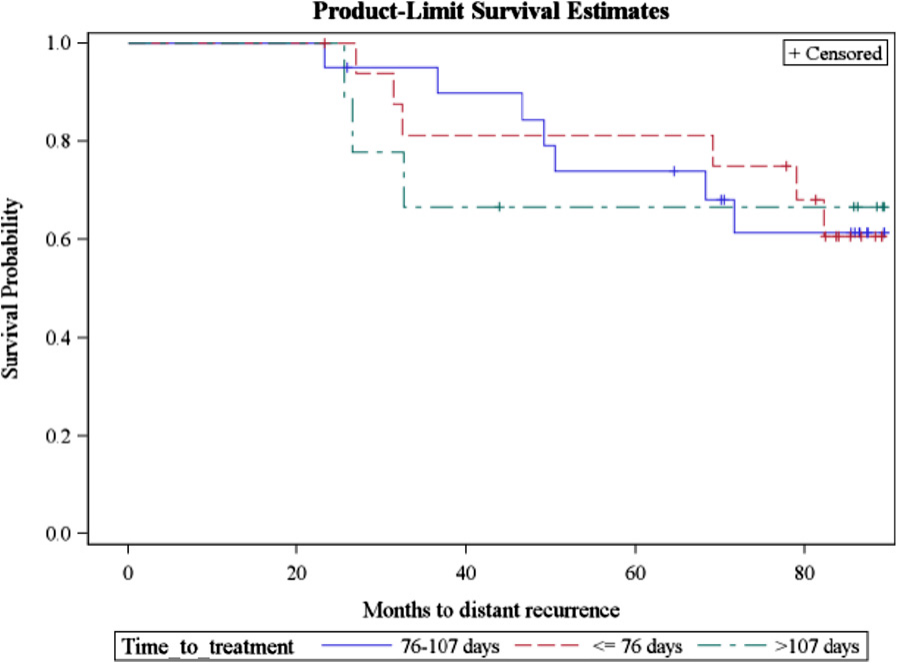
Kaplan Meier curves of time to distant metastases (comparison of 3 groups of time to treatment).

The risk probability of distant recurrence in the next period is associated with an increase of 7.4% (HR = 1.074 (1.0124-1.125), p = 0.0031) for every day that the delivery of RT is extended (Figure 2) and associated with an increase of 2.3% (HR = 1.023 (0.999-1.048), p = 0.0598) for each day that the duration between surgery and initiation of RT is prolonged (Table 3, Figure 2).

**Table 3:** Distant metastases (Cox regression analysis)

**Parameter**	**P value**	**Hazard ratio**	**95% hazard ratio confidence limits**	
Duration of RT days	0.0031	1.074	1.024	1.125
Waiting Time	0.0598	1.023	0.999	1.048

Using logistic regression analysis, we detected a statistically significant increased odds ratio in local recurrence as a function of overall treatment time. Specifically, every day of prolongation of radiotherapy beyond 36 days was associated with an increased risk of local recurrence by 10% (OR = 1.1, p = 0.015). The odds ratio of distant metastases as a function of waiting time was 10% (p = 0.035).

In logistic regression analysis there was no statistically significant correlation between the waiting time and rate of local recurrence (OR- 1,037, p = 0.169) and the overall RT time and rate of distant metastases (OR- 1.024, p = 0.42).

Age, number of lymph nodes (grouped to 3 categories according to nodule staging of TNM) and stage itself do not affect the HR of local recurrence (p = 0.54, 0.99, 0.83 respectively).

It appears that age does not influence the HR of distant metastases (HR 0.99, p = 0.83).

Univariate analysis of the influence of the number of involved lymph nodes and stage on the rate of distant metastases showed the interesting observations. N3 stage (15 or more lymph nodes) compared with lesser nodule stage is linked with HR of 7.2 (p = 0.0011) in association with waiting time to initiation of radiation therapy. Stage 3 of the disease compared with one and two in association with overall treatment time seems to increase the risk of distant metastases (HR = 2.7, p = 0.056).

On multivariate analysis the effect of the overall treatment time and waiting time to initiation of radiation therapy remains strong after adjusting to the number of the involved lymph nodes and stage of the disease (HR = 1.09, p = 00006 and HR = 1.034, p = 0.004, respectively) (Table 4).

**Table 4:** Univariate and multivariate analysis of risk factors for local recurrence and distant metastases

***HR of risk factors for LR and DM (univariate cox regression analysis)***			
Factor	HR for LR (95% CI)	HR for DM (95% CI)	
Time to treatment (Days)	1.038 (0.99, 1.09)	1.023 (1.0,1.04)	
Age at diagnosis	0.98 (0.91, 1.05)	0.996 )0.96,1.04)	
LN group (3 vs (1 + 2))	1 (0,0)	7.183 (2.2,23.4)	
Stage (3 vs 1 + 2)	1.081 (0.11,10.40)	2.703 (0.97,7.5)	
Overall treatment time (Days)	1.101 (1.04,1.17)	1.074 (1.02,1.13)	
***Multivariate analysis of risk factors for DM***			
Factor	HR (95% CI)	Factor	HR (95% CI)
Overall treatment time (Days)	1.090 (1.04,1.14)	Time to treatment (Days)	1.03 (1.00,1.07)
LN group (3 vs (1 + 2))	5.093 (1.12,23.25)	LN group (3 vs (1 + 2))	5.17 (1.12,23.92)
Stage (3 vs 1 + 2)	1.72 (0.43,6.94)	Stage (3 vs 1 + 2)	1.14 (0.29,4.43)

## Discussion

In recent years, two standards have emerged in the adjuvant management of gastric cancer. Cunningham et al. reported that a pre-operative regimen of Epirubicin, Cisplatin and 5-Fluoruracil improves outcome in comparison to patients managed with surgery alone [11]. The results of the INT 0116 study showed that median overall survival for gastric cancer patients in the surgery-only group was 27 months, as compared with 36 months in the chemoradiotherapy group [10]. Smalley et al. recently published 10-year follow up for living patients participating in the INT 0116 study. Overall survival and RFS continue to demonstrate dramatic benefit for patients who received adjuvant radiochemotherapy. Hazard ratios remains virtually unchanged from the initial report [12]. In the original report of INT 0116 there was no mention of prolonged overall treatment time that was attributable to toxicity. We are aware of no published secondary analyses of INT 0116 that describe the impact of temporal factors upon outcome. In fact, despite the recent trend to study the impact of such factors upon outcomes of radiation treatment for a range of malignancies, we were unable to identify any primary papers (in a comprehensive search of databases from PubMed, EMBASE, MEDLINE, and the Cochrane Database) that assessed this parameter for adenocarcinoma of the stomach.

In most radiobiological models [1] it is axiomatic that protracting the course of radiation increases clonogen regeneration in human tumors. The effect of the overall treatment time on local control and distant metastases had been well-studied in several malignant diseases that require irradiation as the primary modality [3,4,8].

The phenomenon of delays in initiating radiation treatment has also been widely discussed. Wyatt RM et al. showed that delays in the initiation of radiotherapy decrease tumor control for head and neck as well as breast cancers. Pursuant to surgery, the growth rates in the residual tumor are maximal due to a relatively high proportion of oxic cells and a reduced cell loss factor. In head and neck tumors, there may be a reduction in local tumor control of up to 1.5% per week’s delay following surgery. Similarly, for breast cancers treated following surgery, there may be a reduction in tumor control of between 0.3% and 1.4% per week’s delay [5]. Do et al. [13] described a 2% decrease in overall survival per day associated with delays in the initiation of radiation treatment for glioblastoma multiforme. Fortin [14] noted a decrease in survival of patients with early-stage head-and-neck cancer if RT was initiated 40 days after the diagnosis. Longer radiotherapy waiting times were found to be associated with diminished survival outcomes for patients treated with definitive radiotherapy for cervix cancer [15].

In contrast, there is evidence that the length of the period prior to the initiation of irradiation does not significantly impair the results of treatment. For instance, Barton et al. concluded, after performing a multivariate analysis of factors pertaining to 581 patients, that waiting time for radiotherapy for early larynx cancer is not associated with increased local recurrence [7]. Similarly, Brouha et al. did not find a correlation between waiting time and the outcome of early glottic cancer [8].

Blumenthal and colleagues presented counter-intuitive findings implying a direct correlation between survival and *prolongation* of the interval between surgery and initiation of irradiation. This anomaly was attributed to insufficient time for recovery following surgically-induced brain trauma [2].

The results of our study showed an association between total treatment time and, to some extent, the time between the surgery and radiation on local control and distant metastases. Modern radiotherapy techniques (e.g., IMRT, IGRT) are designed to optimize dose deposition within the target and to simultaneously spare adjacent normal tissues. Such technologies may have a favorable impact on not only chronic but also acute symptoms. If indeed radiotherapy is tolerated with a minimum of acute morbidity, then it is likely that overall treatment time will not be prolonged thereby improving local and distant control of disease. It must be acknowledged that one of the limitations in such a retrospective analysis could be the fact that patients who were “sicker” suffered more acute morbidity and that the decrements in local and distant control were actually a function of their inherent illness as opposed to an outgrowth of temporal delays in radiation delivery.

We also observed a correlation between the time from the surgery to the initiation of radiation therapy and the rate of distant metastases (HR 1.02, p- 0.059), that is extremely prominent in patients with locally advanced disease. This finding seems to offer an impetus to shorten the post-op interval prior to initiating adjuvant irradiation. Issues related to the impact of patient recovery following surgery are beyond the scope of this report.

There are, of course, multiple limitations of this retrospective study of a relatively small number of patients and accordingly, the findings are more exploratory than conclusive. Multivariate analysis of the impact of different co-variants on the endpoints of local control, freedom from distant metastases and even overall survival among gastric cancer patients is wanting in the scientific literature. Nevertheless physicians should be cognizant of the potential influence of the temporal factors discussed and therefore are advised to redouble efforts to maximize efficiencies in delivering treatment without unnecessary delays and interruptions.

## Competing interests

All authors have no competing interest to disclose. There are no financial disclosures from any author.

## Authors’ contribution

All authors read and approved the final manuscript. VS participated in the design of the study, study coordination, data acquisition, data analysis, drafting the manuscript. RG participated in the design of the study, acquisition of the data, revised the manuscript critically for intellectual content. MM participated in acquisition of the data, data analysis and revised the manuscript critically for intellectual content. MI participated in the data analysis, study coordination and revised the manuscript critically for intellectual content. ESS participated in study coordination, data analysis and revised the manuscript critically for intellectual content. BWC participated in the design of the study, acquisition and interpretation of the data, drafting the manuscript and revised the manuscript critically for intellectual content.
